# β-catenin regulates the formation of multiple nephron segments in the mouse kidney

**DOI:** 10.1038/s41598-019-52255-w

**Published:** 2019-11-04

**Authors:** Patrick Deacon, Charles W. Concodora, Eunah Chung, Joo-Seop Park

**Affiliations:** 1 Division of Pediatric Urology and Division of Developmental Biology, Cincinnati Children’s Hospital Medical Center, Cincinnati, OH 45229 USA; 2 University of Cincinnati College of Medicine, Cincinnati, OH 45267 USA; 3 Present Address: Urology for Children, 200 Bowman Drive, Voorhees, NJ 08043 USA

**Keywords:** Differentiation, Organogenesis, Mesoderm

## Abstract

The nephron is composed of distinct segments that perform unique physiological functions. Little is known about how multipotent nephron progenitor cells differentiate into different nephron segments. It is well known that β-catenin signaling regulates the maintenance and commitment of mesenchymal nephron progenitors during kidney development. However, it is not fully understood how it regulates nephron segmentation after nephron progenitors undergo mesenchymal-to-epithelial transition. To address this, we performed β-catenin loss-of-function and gain-of-function studies in epithelial nephron progenitors in the mouse kidney. Consistent with a previous report, the formation of the renal corpuscle was defective in the absence of β-catenin. Interestingly, we found that epithelial nephron progenitors lacking β-catenin were able to form presumptive proximal tubules but that they failed to further develop into differentiated proximal tubules, suggesting that β-catenin signaling plays a critical role in proximal tubule development. We also found that epithelial nephron progenitors lacking β-catenin failed to form the distal tubules. Expression of a stable form of β-catenin in epithelial nephron progenitors blocked the proper formation of all nephron segments, suggesting tight regulation of β-catenin signaling during nephron segmentation. This work shows that β-catenin regulates the formation of multiple nephron segments along the proximo-distal axis of the mammalian nephron.

## Introduction

The mammalian nephron is composed of multiple segments^1,2^. Podocytes and the Bowman’s capsule in the renal corpuscle are followed by the proximal tubule (PT), loop of Henle (LOH), and distal tubule (DT)^1,2^. Each of these segments performs unique physiological functions, indicating that different cell types are present in different nephron segments^3,4^. All nephron tubule cells are derived from the multipotent mesenchymal nephron progenitors (MNPs) which are located at the cortex of the developing kidney^5,6^. MNP undergo mesenchymal-to-epithelial transition (MET), forming the renal vesicle (RV). Epithelial nephron progenitors in the RV undergo complex morphogenesis to form the S-shaped body (SSB) which eventually develops into a nephron^2^ (Figure S1).

Little is known about which signaling pathways direct nephron progenitors to develop into distinct nephron segments. It had been reported that, in the mouse kidney, Notch signaling promotes the formation of PT and represses the formation of DT^7–9^. However, we have recently shown that Notch signaling regulates the formation of all nephron segments in the mouse kidney without preferentially promoting the formation of a specific segment^10,11^. It was shown that, in the zebrafish pronephros, proximo-distal segmentation was regulated by retinoic acid signaling rather than by Notch signaling^12,13^. It has yet to be determined if retinoic acid signaling regulates the mammalian nephron in a similar manner.

β-catenin plays an essential role in mediating canonical Wnt signaling^14^. Binding of a Wnt ligand to a Frizzled receptor and a co-receptor Lrp5/6 results in the accumulation of β-catenin in the cytosol and eventually in the nucleus. β-catenin forms a complex with a member of the Tcf family transcription factors to regulate expression of the target genes. Loss-of-function (LOF) and gain-of-function (GOF) mutations of β-catenin have been powerful tools for investigating canonical Wnt signaling *in vivo*^15^. A potential caveat is that the resulting phenotypes may be, at least in part, due to defective cell adhesion since β-catenin is known to form a complex with cadherin at the plasma membrane^16^.

It is now well established that Wnt/β-catenin signaling is required for the maintenance and early differentiation of MNPs^17–21^. We have previously shown that MNPs lacking β-catenin fail to form RV^17^. However, due to this early developmental arrest in nephrogenesis, this study provided little information about if or how Wnt/β-catenin signaling regulates epithelial nephron progenitors (e.g. RV and its derivatives) to develop into the mature nephron. Several studies have shown that Wnt/β-catenin signaling is important at later stages of nephron development. When *Ctnnb1*, the gene encoding β-catenin, was deleted using *Pax8Cre* which targets both the developing nephron and collecting duct (CD) in the mouse kidney, formation of the Bowman’s capsule was defective^22^. In addition, based on the pharmacological manipulation of Wnt/β-catenin signaling in mouse kidney explants, it was proposed that Wnt/β-catenin signaling promotes the formation of distal segments of the nephron and represses the formation of proximal segments^23^. Taken together, these studies suggest that Wnt/β-catenin signaling may continuously regulate mammalian nephron development even after β-catenin-triggered epithelialization of MNPs.

In order to investigate how β-catenin signaling regulates mammalian nephron development in epithelial nephron progenitors, we have performed genetic analyses of β-catenin by specifically targeting the developing nephron in the mouse kidney. Here, we report that epithelial nephron progenitor cells lacking β-catenin can form presumptive PT cells but cannot form differentiated PT cells. We also find that β-catenin is required for the formation of DT. In summary, our data suggest that β-catenin signaling is essential for the development and maturation of multiple nephron segments in the mammalian kidney.

## Results

### Lineage analysis with *Osr2Cre* in the developing mouse kidney

**Figure 1:**
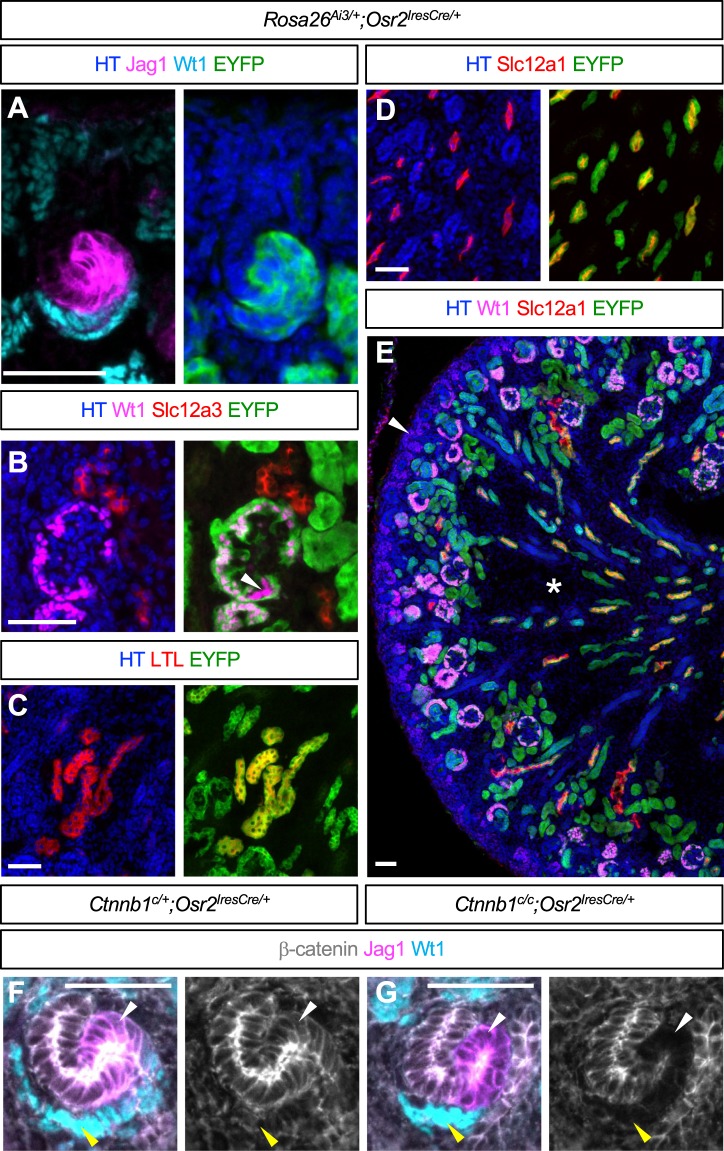
(**A**–**E**) Lineage analysis with *Osr2Cre* in the developing mouse kidney. Cre-mediated recombination activates expression of EYFP reporter, which labels *Osr2*-expressing cells and their descendants. (**A**) *Osr2Cre* targets the proximal (Wt1+) and medial (Jag1+) segments of the S-shaped body. In the nephron, *Osr2Cre* targets podocytes (**B**), proximal tubules (**C**), and loops of Henle (**D**), but not distal tubules (**B**). White arrowhead in (**B**) points to podocytes that escaped *Osr2Cre*. (**E**) From the absence of EYFP signal (green), we conclude that *Osr2Cre* targets neither the cap mesenchyme (white arrowhead, for example) nor the interstitial cells (asterisk). (**F**) β-catenin is ubiquitously expressed in the S-shaped body. (**G**) *Osr2Cre* removes β-catenin from the proximal (yellow arrowhead) and medial (white arrowhead) segments of the S-shaped body. Images are representative of three independent experiments. HT, Hoechst. Stage E18.5. Scale bar: 50 μm.

In order to investigate the role of β-catenin signaling in mammalian nephron segmentation, we set out to perform β-catenin LOF studies, specifically targeting the epithelial nephron progenitors in the mouse kidney. Since β-catenin is ubiquitously expressed in the kidney^4^, the specificity of Cre is important. *Wnt4GFPcre* (*Wnt4*^*tm3(EGFP/cre)Amc*^ or *Wnt4*^*tm2(EGFP/cre)Svo*^)^24,25^ and *Pax8Cre* (*Pax8*^*tm1(cre)Mbu*^)^26^ are widely used to target nephron tubules. However, the expression of these Cre lines is not exclusive to the developing nephron tubules; *Wnt4GFPcre* also targets the medullary stroma^27^ and a subset of MNPs^11,28,29^ while *Pax8Cre* targets the collecting duct in addition to the nephron lineage^30^. Removal of β-catenin from these non-nephron tubule cells may indirectly affect nephron segmentation. Therefore, we chose to use *Osr2Cre* (*Osr2*^*tm2(cre)Jian*^) which was previously shown to target developing nephrons in the mouse kidney^31^. To examine when *Osr2Cre* is activated and which nephron segments it targets, we performed lineage analysis employing Cre-mediated activation of a Rosa reporter (Ai3, *Gt(ROSA)26Sor*^*tm3(CAG-EYFP)Hze*^)^32^. We found that *Osr2Cre* labeled mature RV and the comma-shaped body, but not nascent RV, with the Rosa reporter (Figure S2). In the SSB, *Osr2Cre* targeted the proximal and medial segments but not the distal segment (Fig. 1A). To determine which nephron segments these Rosa reporter-positive cells in the SSB develop into, we performed co-immunostaining of EYFP and nephron segmentation markers. We found that the Rosa reporter was active in the podocytes, the Bowman’s capsule, PT, and LOH (Fig. 1B–D) but that it was inactive in the DT (Fig. 1B). Furthermore, we found that, unlike *Wnt4GFPcre* or *Pax8Cre*, *Osr2Cre* did not target MNPs, medullary stroma, or the CD (Fig. 1E). These data showed that *Osr2Cre* specifically targets all nephron segments except for the DT. We noticed that *Osr2Cre* mosaically targeted Wt1+ cells in the nascent nephrons in the nephrogenic zone (Figure S3) but that *Osr2Cre* labeled most of the Wt1+ cells in glomeruli with the Rosa reporter (Figure S3). This result suggests that *Osr2Cre* may not target the proximal and medial segments of the SSB simultaneously.

We generated the β-catenin LOF mutant kidney with *Osr2Cre* and examined the presence of β-catenin in the SSB. In the control kidney, β-catenin was ubiquitously expressed in the SSB (Fig. 1F). Consistent with *Osr2Cre*-mediated activation of the Rosa reporter in the SSB (Fig. 1A), we found that, in the β-catenin LOF mutant kidney, the proximal and medial segments of the SSB contained considerably less β-catenin (Fig. 1G). Removal of β-catenin from the proximal segment of SSB was comparable to that from the medial segment. This allowed us to investigate how β-catenin regulates the development of SSB into the proximal nephron segments including podocytes, PT, and LOH.

### β-catenin is required for the proper formation of the renal corpuscle

**Figure 2:**
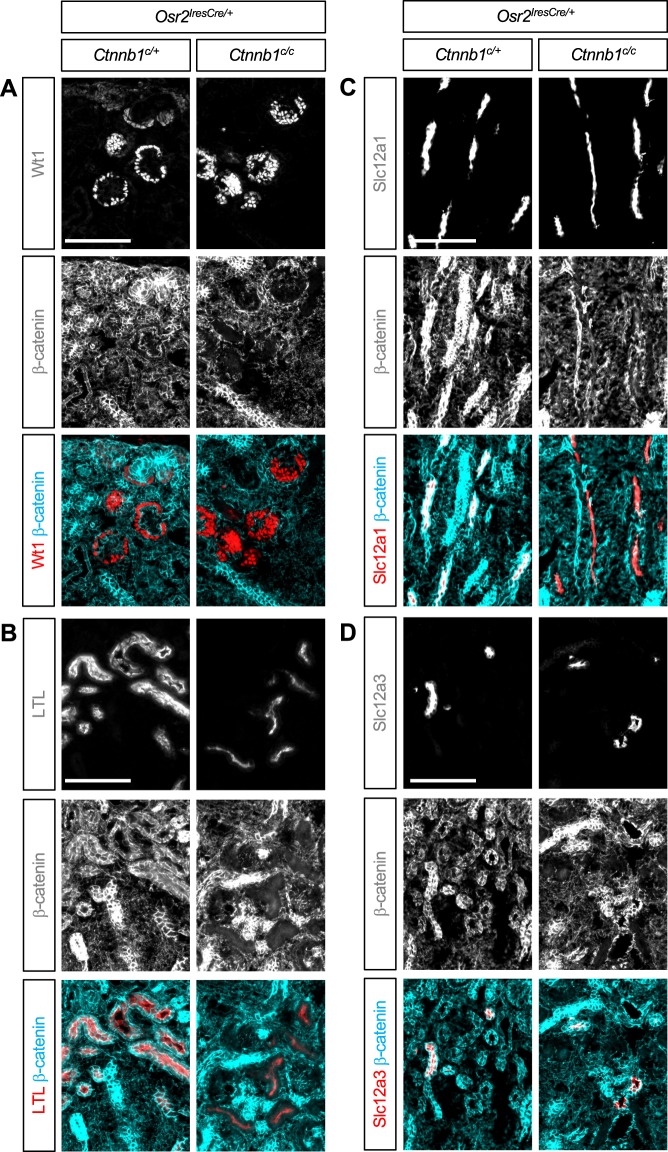
(**A**–**C**) In the β-catenin loss-of-function mutant kidney by *Osr2Cre*, the podocytes, proximal tubules, and loops of Henle show little β-catenin staining, suggesting that *Ctnnb1*, the gene encoding β-catenin, was deleted by *Osr2Cre*. Cells lacking β-catenin are able to form Wt1+ podocytes (**A**), LTL + proximal tubules (**B**), and Slc12a1+ loops of Henle (**C**). Note, however, that the β-catenin mutant kidney shows aberrant arrangement of Wt1+ podocytes, weaker LTL staining, and thinner Slc12a1+ tubules, suggesting developmental defects. (**D**) In the β-catenin mutant kidney, β-catenin is still present in Slc12a3+ distal tubules, consistent with the fact that *Osr2Cre* does not target distal tubules. Images are representative of three independent experiments. Stage E18.5. Scale bar: 100 μm.

To determine how loss of β-catenin in the SSB affects the development of nephron segments, we examined nephron segmentation markers in the β-catenin LOF mutant kidney with *Osr2Cre* (*Ctnnb1*^*c/c*^*;Osr2*^*IresCre*/+^) and its control (*Ctnnb1*^*c*/+^*;Osr2*^*IresCre*/+^) kidney. We used Wt1, *Lotus tetragonolobus* lectin (LTL), and Slc12a1 to mark podocytes, PT, and LOH, respectively. We found that all of those nephron segments were present in the mutant kidney (Fig. 2A–C), suggesting that the initial specification of the nephron segments occurred in the absence of β-catenin.

To confirm that β-catenin was removed from each nephron segment in the mutant kidney, we examined the expression of β-catenin along with nephron segmentation markers. We found that, in the mutant kidney, these nephron segmentation marker-positive cells contained considerably less β-catenin compared to their counterparts in the control kidney (Fig. 2A–C), showing that the β-catenin gene (*Ctnnb1*) was indeed deleted by *Osr2Cre*. Our results suggest that β-catenin is dispensable for the initial specification of podocytes, PT, and LOH during nephron development. We also found that Slc12a3+ DT cells were present in the mutant kidney (Fig. 2D) but, since the DT cells were not targeted by *Osr2Cre* (Fig. 1B), these cells were still positive for β-catenin in the mutant kidney (Fig. 2D).

**Figure 3:**
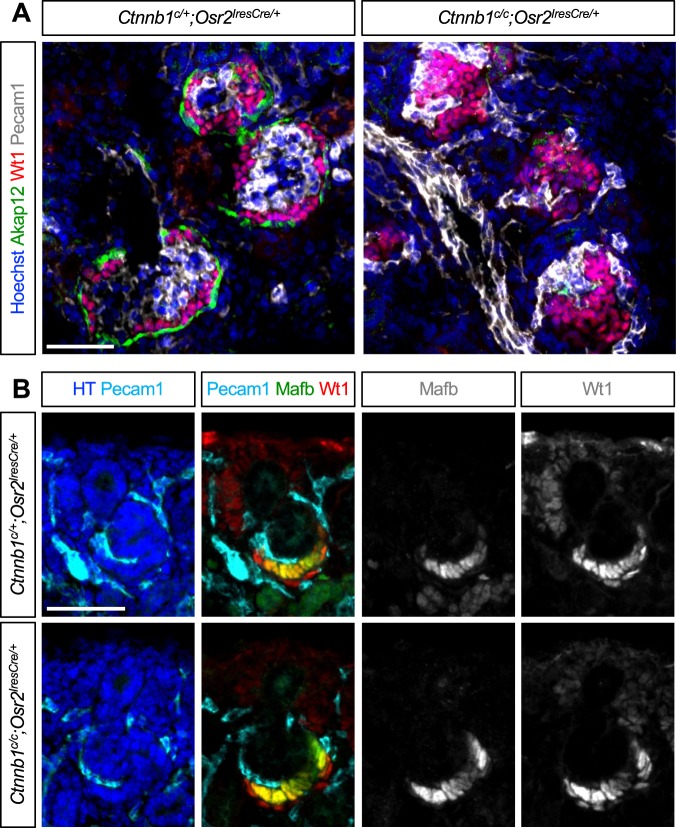
β-catenin is required for the proper formation of the renal corpuscle. (**A**) In the renal corpuscle of the control kidney, Wt1+ podocytes surround Pecam1+ endothelial cells in a crescent configuration and Akap12 marks parietal epithelial cells. By contrast, in the β-catenin mutant kidney, Pecam1 + endothelial cells fail to populate inside the renal corpuscle and Akap12 expression is considerably reduced. (**B**) Pecam1 + endothelial cells invade the vascular cleft in the S-shaped body in both control and mutant kidneys. In the proximal segment of the S-shaped body, Wt1 is detected in both visceral and parietal epithelial cells while Mafb is detected in the visceral epithelial cells only. The initial specification of visceral and parietal epithelial cells appears normal in the β-catenin mutant kidney. Images are representative of three independent experiments. HT, Hoechst. Stage E18.5. Scale bar: 50 μm.

We observed a defective formation of the renal corpuscle in the β-catenin LOF mutant kidney by *Osr2Cre*. In the control kidney, Wt1+ podocytes surrounded Pecam1 + endothelial cells in the renal corpuscle (Fig. 3A). However, in the β-catenin mutant kidney, endothelial cells failed to populate inside the renal corpuscle and podocytes failed to form the single cell layered crescent configuration characteristic of a normal renal corpuscle (Fig. 3A). In addition, we found that expression of *Akap12*, a marker for parietal epithelial cells (also known as SSeCKS)^33^, was considerably reduced in the mutant kidney. This result is consistent with a previous report, which showed that removal of β-catenin in renal epithelial cells by *Pax8Cre* causes defects in the gross architecture of glomeruli^22^.

To assess if the aberrant formation of the renal corpuscle seen in the β-catenin LOF mutant kidney by *Osr2Cre* is caused by a defect in the early specification of visceral epithelial cells (presumptive podocyte progenitors) or parietal epithelial cells (presumptive Bowman’s capsule progenitors), we examined the SSB stage. We found that, in both control and mutant SSBs, endothelial cells migrated into the vascular cleft which was formed by the proximal and medial segments of the SSB, suggesting that β-catenin is dispensable for the visceral epithelial cells to recruit endothelial cells (Fig. 3B). In the proximal segment of the SSB, the visceral and parietal epithelial cells showed distinct cell morphologies and differential gene expression. The visceral epithelial cells were organized in a columnar manner while the parietal epithelial cells were arranged in a thin end-to-end pattern (Fig. 3B). Furthermore, the visceral epithelial cells expressed *Wt1* and *Mafb*, two genes required for podocyte development^34–36^, while the parietal epithelial cells expressed *Wt1*, but not *Mafb* (Fig. 3B). We found that the visceral and parietal epithelial cells lacking β-catenin showed normal cell morphologies and gene expression in the SSB (Fig. 3B). Our results show that β-catenin is dispensable for the initial specification of visceral and parietal epithelial cells in the SSB.

### β-catenin is required for presumptive proximal tubules to develop into differentiated proximal tubules

**Figure 4:**
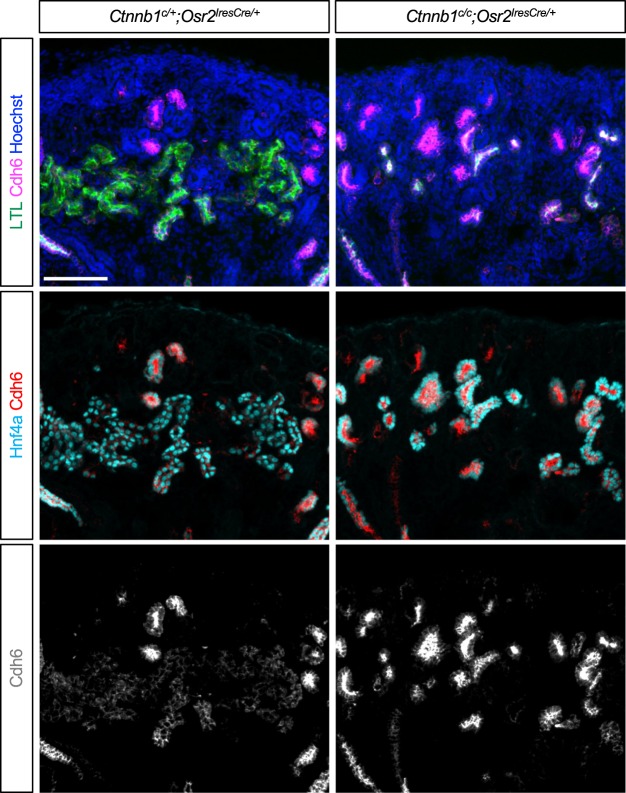
β-catenin is required for the formation of differentiated proximal tubule cells with strong LTL staining. Hnf4a marks both presumptive and differentiated proximal tubules. In the control kidney, presumptive proximal tubules show strong Cdh6 signal and weak LTL staining while differentiated proximal tubules show weak Cdh6 signal and strong LTL staining. In the β-catenin loss-of-function mutant kidney by *Osr2Cre*, all Hnf4a+ cells show strong Cdh6 signal, failing to form differentiated proximal tubules with strong LTL staining. Images are representative of three independent experiments. Stage E18.5. Scale bar: 100 μm.

Previously, we have shown that, in the developing mouse kidney, presumptive PT cells show weak LTL staining while differentiated PT cells show strong LTL staining and that presumptive PT cells lacking *Hnf4a*, a gene encoding a transcription factor specifically expressed in PT cells in the kidney, fail to develop into differentiated PT cells^37^. We found that the PT cells present in the β-catenin LOF mutant kidney showed substantially weaker LTL staining compared to the PT cells found in the control kidney (Fig. 2B). Since weak LTL staining of PT cells in the β-catenin LOF mutant kidney was reminiscent of the phenotype seen in the *Hnf4a* mutant kidney, we examined if presumptive PT cells in the β-catenin LOF mutant kidney failed to develop into differentiated PT cells. It has been reported that *Cdh6* is expressed in presumptive PT cells and that its expression is downregulated in LTL-stained PT cells, suggesting that Cdh6 marks PT progenitor cells^38^. Consistent with this, we found an inverse correlation between *Cdh6* expression and LTL staining in the control kidney (Fig. 4). In addition, we found that both Cdh6-positive cells and LTL-stained cells expressed Hnf4a, a transcription factor specifically expressed in the PT cells^3^ and required for PT development^37^. Downregulation of *Cdh6* was accompanied by robust LTL staining and increased distance between Hnf4a+ nuclei, suggesting the maturation and enlargement of PT cells (Fig. 4). Collectively, these observations suggest that Cdh6 is a marker for presumptive PT cells. We found that, in the β-catenin LOF mutant kidney, most of the Hnf4a+ cells showed strong Cdh6 signal and shorter internuclei distance, indicating that they are presumptive PT cells (Fig. 4 and Figure S4A). The mutant kidney failed to form Hnf4a+ cells with strong LTL staining nor do they express *Slc5a2*, a mature PT marker gene encoding a sodium-glucose cotransporter (Figure S4B), suggesting that presumptive PT cells lacking β-catenin fail to develop into differentiated PT cells. Our results show that β-catenin is required for presumptive PT cells to further develop into differentiated PT cells.

### β-catenin is required for the formation of the distal tubules

Since *Osr2Cre* does not target the DT cells, it does not allow us to investigate how Wnt/β-catenin signaling regulates the formation of DT. To address this, we generated the β-catenin LOF mutant kidney (*Ctnnb1*^*c/c*^*;Wnt4*^*GFPcre*/+^) using *Wnt4GFPcre* (*Wnt4*^*tm3(EGFP/cre)Amc*^)^24^. We have previously shown that *Wnt4GFPcre* can target the nephron lineage including DT^11^ but since *Wnt4GFPcre* also targets the medullary stroma^27^, *Wnt4GFPcre* may not be suitable for studying the role of β-catenin in LOH because removal of β-catenin in the medullary stroma may affect the development of the adjacent LOH^39^. However, this is less of a concern for studying the DT because the DT cells are located close to the cortical region of the developing mouse kidney, away from the medullary stroma.

**Figure 5:**
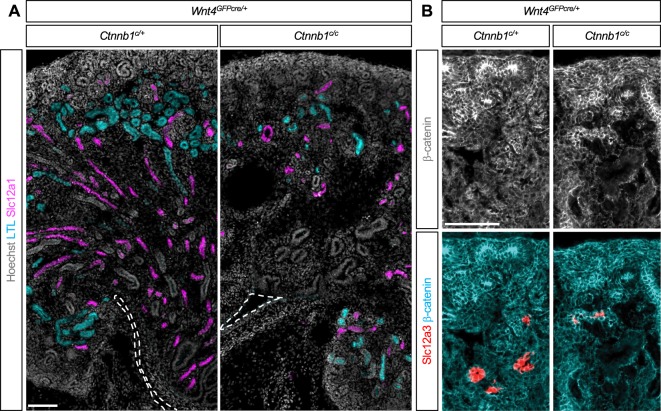
Removal of β-catenin by *Wnt4Cre* inhibits loop of Henle elongation and distal tubule formation. (**A**) Slc12a1+ cells in the control kidney elongate, contributing to papilla formation. Slc12a1+ cells in the β-catenin loss-of-function mutant kidney by *Wnt4Cre* fail to elongate, causing a defective papilla formation. The ureter is marked by broken lines. (**B**) In the β-catenin loss-of-function mutant kidney by *Wnt4Cre*, there are less Slc12a3+ distal tubules and the few that have formed are positive for β-catenin, suggesting that these cells have escaped *Wnt4Cre*-mediated removal of β-catenin. The absence of β-catenin-negative distal tubule cells suggests that β-catenin is required for the formation of distal tubules. Images are representative of three independent experiments. Stage E18.5. Scale bar: 100 μm.

Consistent with our observation that PT development was arrested in the β-catenin LOF mutant kidney by *Osr2Cre* (Fig. 4), the β-catenin LOF mutant kidney by *Wnt4GFPcre* also showed fewer PT cells and weaker LTL staining (Fig. 5A). In the control kidney, Slc12a1+ cells elongated toward the papilla region, forming the characteristic LOH structure. By contrast, in the β-catenin LOF mutant kidney by *Wnt4GFPcre*, Slc12a1+ cells failed to elongate, stunting the development of the papilla (Fig. 5A). Considering the fact that the β-catenin LOF mutant kidney by *Osr2Cre* showed relatively normal LOH formation (Fig. 2A), the failed elongation of LOH seen in Fig. 5A was likely to be caused by removal of β-catenin in the medullary stroma by *Wnt4GFPcre*.

When *Ctnnb1* was deleted with *Wnt4GFPcre*, we found that the mutant kidney formed considerably fewer DT cells as marked by Slc12a3, a DT-specific transporter^3^ (Figure S5). Interestingly, the few detectable DT cells found in the mutant kidney were positive for β-catenin (Fig. 5B and Figure S5), suggesting that these cells had escaped *Wnt4GFPcre*-mediated removal of β-catenin. The absence of β-catenin-negative, Slc12a3-positive DT cells in the mutant kidney suggests that β-catenin signaling is required for the formation of the DT.

### Expression of a stable form of β-catenin in the developing nephron blocks proper nephron segmentation

Our β-catenin LOF studies suggest that β-catenin regulates the development of multiple nephron segments in epithelial nephron progenitors. To further explore the mechanism of β-catenin-mediated nephron development, we performed a β-catenin GOF study using *Wnt4GFPcre* or *Osr2Cre*. The conditional allele used in this study has the third exon of the *Ctnnb1* gene flanked by two *LoxP* sites (*Ctnnb1*^*ex3*^)^40^. Since the third exon encodes the N-terminus of β-catenin protein, Cre-mediated recombination results in the production of an N-terminally truncated β-catenin which is resistant to degradation. Increased abundance of β-catenin leads to the activation of canonical Wnt signaling in a ligand-independent manner^40^.

**Figure 6:**
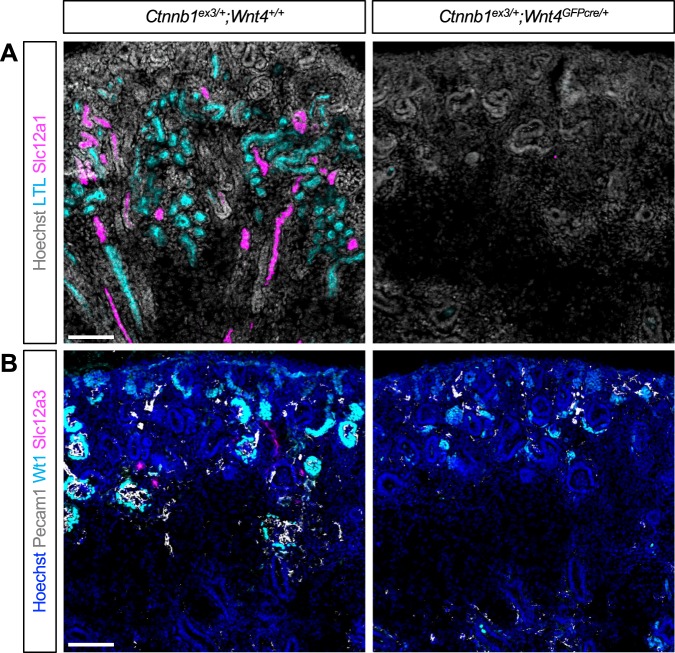
Expression of a stable form of β-catenin inhibits proper nephron patterning. The β-catenin gain-of-function mutant kidney by *Wnt4GFPcre* fails to form properly patterned nephrons. (**A**) No proximal tubules or Slc12a1+ loops of Henle are formed in the mutant kidney. (**B**) No glomeruli or Slc12a3+ distal tubules are formed in the mutant kidney. Images are representative of three independent experiments. Stage E18.5. Scale bar: 100 μm.

We found that *Wnt4GFPcre*-mediated activation of a stable form of β-catenin blocked the formation of all nephron segments (Fig. 6). No glomeruli, PT, LOH, or DT were found in the mutant kidney. Similarly, we found that the β-catenin GOF mutant kidney by *Osr2Cre* lacked glomeruli and PTs (Figure S6). Slc12a1+ LOH cells and Slc12a3+ DT cells were present in the β-catenin GOF mutant kidney but they failed to elongate or form normal LOH or DT segments (Figure S6). Given that *Osr2Cre* does not target DT, it was unexpected that Slc12a3+ DT cells were reduced in this mutant (see Discussion). We found no evidence that constitutive activation of Wnt/β-catenin signaling in the developing nephrons promoted the formation of any specific nephron segment. Instead, we found that expression of a stable form of β-catenin in the developing nephron inhibited the formation of all nephron segments.

**Figure 7:**
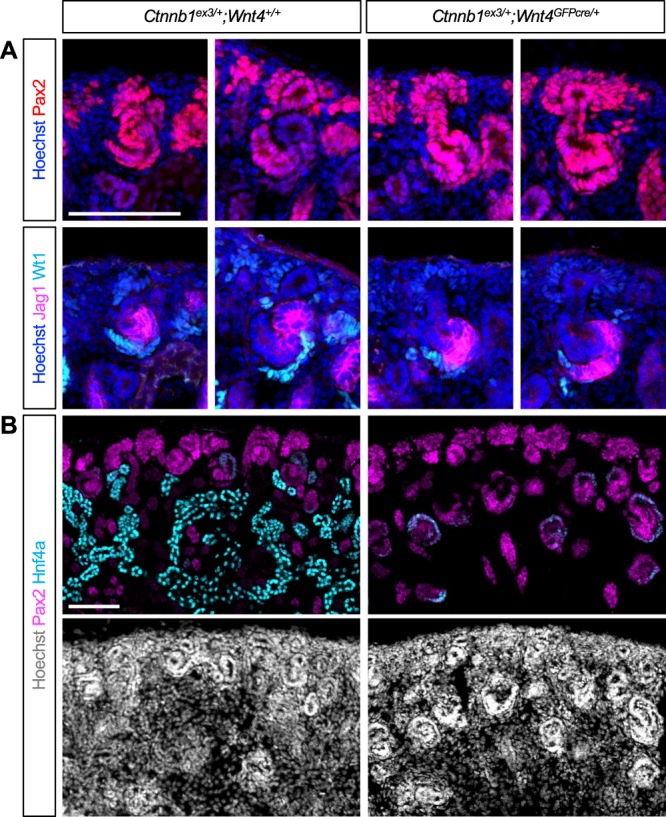
Expression of a stable form of β-catenin prevents epithelial nephron progenitors from further differentiating. (**A**) Nascent (left) and mature (right) S-shaped bodies are shown. In the control kidney, Jag1 and Wt1 mark the medial and proximal segments of the S-shaped body, respectively. In the β-catenin gain-of-function mutant kidney by *Wnt4GFPcre*, the Jag1 expression domain expands into the proximal segment of the S-shaped body. Pax2 marks the collecting duct, the cap mesenchyme, and nascent developing nephrons. (**B**) In the control kidney, Pax2 expression in epithelial nephron progenitors is downregulated after S-shaped body stage. As a result, most of the Hnf4a+ cells are negative for Pax2. In the β-catenin gain-of-function mutant kidney, Pax2 is persistently expressed in the nephron lineage and all Hnf4a+ cells are also positive for Pax2, suggesting that β-catenin gain-of-function mutant cells fail to exit from their progenitor status. Images are representative of three independent experiments. Stage E18.5. Scale bar: 100 μm.

To investigate how expression of a stable form of β-catenin interferes with nephron segmentation, we examined early development of the nephron in the β-catenin GOF mutant kidney by *Wnt4GFPcre*. In the control kidney, the proximal and medial segments of the SSB were marked by Wt1 and Jag1, respectively, and the expression domains of Wt1 and Jag1 did not overlap, showing clear separation (Fig. 7A). By contrast, in the β-catenin GOF mutant kidney, we observed an overlap of the Wt1 expression domain and the Jag1 expression domain. Although the initial morphology of the SSB appeared relatively normal in the β-catenin GOF mutant kidney, the Jag1 expression domain expanded aberrantly toward the Wt1 expressing proximal segment of the SSB (Fig. 7A). This result shows that the β-catenin GOF mutant kidney by *Wnt4GFPcre* exhibits a defect in nephron segmentation as early as the SSB stage. We observed a similar phenotype in the β-catenin GOF mutant kidney by *Osr2Cre* (Figure S7A).

One of the most striking defects seen in the β-catenin GOF mutant kidney by *Osr2Cre* or *Wnt4GFPcre* was the complete lack of LTL + PTs (Fig. 6A and Figure S6). Since Hnf4a plays a critical role in PT development^37^, we examined Hnf4a expression in the β-catenin GOF mutant kidney. In the nephron lineage of the control kidney, Pax2 expression was mostly restricted to the nephrogenic zone at the cortex, marking the cap mesenchyme and nascent developing nephrons (Fig. 7B). In the control kidney, Pax2 was undetectable in most of the Hnf4a+ PT cells, suggesting that the formation of PTs is accompanied by downregulation of Pax2 (Fig. 7B). Interestingly, we found that, in the β-catenin GOF mutant kidney by *Wnt4GFPcre*, all Hnf4a+ cells were positive for Pax2 (Fig. 7B). Pax2 expression was not restricted to the nephrogenic zone in the mutant kidney. Instead, most of the epithelial structures found in the mutant kidney showed persistent expression of Pax2 (Fig. 7B), suggesting that epithelial nephron progenitors experiencing persistent β-catenin activity are developmentally arrested, failing to further differentiate into PTs. Taken together, our results suggest that tight regulation of β-catenin signaling is required for the proper patterning of the nephron even after initial commitment of nephron progenitors. We observed a similar phenotype in the β-catenin GOF mutant kidney by *Osr2Cre* (Figure S7B).

## Discussion

β-catenin is known to regulate the maintenance and commitment of the MNPs^17–21^. Here we show that it continues to play critical roles in nephron development after the initial commitment of nephron progenitors. Strikingly, we found that, in epithelial nephron progenitors, β-catenin signaling regulates the development of multiple nephron segments without promoting the formation of any specific segment.

Little is known about how multipotent MNPs develop into different nephron segments during kidney development^1,2^. The first sign of nephron segmentation can be seen as early as the renal vesicle (RV) stage^24,38,41,42^. The distal part of the RV (the part closer to the branching tip of the collecting duct) shows differential gene expression compared to the rest of the RV (Figure S1A). We have previously identified multiple β-catenin-bound enhancers that are associated with genes activated during differentiation of MNPs^19^. Transgenic analyses in the developing mouse kidney revealed that these enhancers were active in the distal RV but inactive in the proximal RV^19^. In addition, we have previously shown that the expression of *Jag1* in the distal RV was correlated with the downregulation of *Six2* while Six2 was still detectable in the proximal RV^10^. The cellular identity of the MNPs appears closer to that of the proximal RV than that of the distal RV, consistent with a recent finding that the cells in the proximal RV exit from the cap mesenchyme later than the cells of the distal RV^43^.

By the time the RV is transformed into the SSB, three discrete (proximal, medial and distal) segments are established^2^ (Figure S1A). The proximal segment of the SSB is thought to develop into the renal corpuscle because it expresses podocyte marker genes such as *Wt1* and *Mafb* (Fig. 3B). The distinct cell morphologies of the two cell layers in this segment suggest that the visceral and parietal epithelial cells develop into podocytes and Bowman’s capsule, respectively. The fact that *Osr2Cre* targets the medial segment, but not the distal segment, of the SSB helped us reveal that the medial segment develops into PT and LOH. This is consistent with our previous report that Hnf4a, a transcription factor required for the PT development, was detected in the proximal subset of the medial segment of the SSB^37^ and suggests that Hnf4a-negative cells in the distal subset of the medial segment develop into LOH. This then allowed us to infer that the distal segment of the SSB develops into the DT. Our lineage analysis with *Osr2Cre* uncovered a correlation between SSB segments and mature nephron segments.

Lindström *et al*. have suggested that a gradient of β-catenin activity regulates segmentation in the nascent nephron^23^. They manipulated Wnt/β-catenin signaling pharmacologically in the embryonic kidney explant which is suitable for studying early nephrogenesis rather than the formation of mature nephron segments. They mainly focused on how β-catenin regulates the formation of different segments in the SSB. It is unclear how the altered segmentation of the SSB influences the segmentation of the fully formed nephron. Unlike the pharmacological approach which targets every cell in the kidney explant *in vitro*, our genetic approach allowed us to specifically target epithelial nephron progenitors and their descendants *in vivo*. Although *Osr2Cre* is active in the mature RV (Figure S2), one has to consider that there is lag time between Cre-mediated recombination and the intended effects; degradation of *Ctnnb1* mRNA and β-catenin protein must occur in order to achieve removal of β-catenin while the production of a stable form of β-catenin requires transcription and translation after Cre removes the third exon of *Ctnnb1*. Hence, our genetic approach may not be suitable for studying the role of β-catenin on the transformation of RV into SSB. Since the initial formation of SSB appears relatively normal in both β-catenin loss-of-function and gain-of-function mutant kidneys, we think that the effects of genetic manipulation of β-catenin reported in this work mainly reflects how β-catenin regulates the development of SSB into mature nephron segments.

Since the β-catenin LOF mutants by *Osr2Cre* die shortly after birth with severe craniofacial defects^44^, we could not analyze the mutant kidney postnatally. By contrast, the β-catenin LOF mutants by *Pax8Cre* show no lethality until 2 weeks of age^22^. In the kidney, *Pax8Cre* targets DT and CD in addition to the nephron segments targeted by *Osr2Cre*. Previous electron microscopy analysis of the β-catenin LOF mutant kidney by *Pax8Cre* showed a glomerulogenesis defect similar to our results shown in Fig. 3A. Furthermore, it was reported that the β-catenin LOF mutant adult kidney by *Pax8Cre* lacks the outermost cortex^22^, implying a similar PT defect seen in our study. However, the effects on other nephron segments were not investigated in that report.

We have previously shown that, in the absence of *Hnf4a*, presumptive PT cells failed to develop into differentiated PT cells, suggesting that Hnf4a is dispensable for the formation of presumptive PT cells but required for the formation of differentiated PT cells^37^. It is quite interesting that removal of β-catenin prevented presumptive PT cells from developing into differentiated PT cells, showing a similar defect seen in the *Hnf4a* LOF mutant kidney. A number of reports suggested a potential interaction between the Wnt/β-catenin pathway and Hnf4a^45–49^. Notably, it was shown that Hnf4a and β-catenin not only form a complex but also share common target genes in the liver^48^. Identification of common target genes regulated by both Hnf4a and β-catenin in presumptive PT cells would help us understand how these two factors coordinate PT development.

We found that the β-catenin LOF mutant kidney developed structures resembling LOH as judged by the expression of *Slc12a1*, a gene encoding a LOH-specific transporter. However, the mutant LOHs were substantially thinner compared to those found in the control kidney (Fig. 2C). Therefore, it is possible that β-catenin regulates the maturation of LOH. Since *Osr2Cre* does not target the DT cells, we generated another β-catenin LOF mutant kidney using *Wnt4GFPcre* which does target DT^11^. We found that all the DT cells formed in the β-catenin LOF mutant kidney were positive for β-catenin (Fig. 5 and Figure S5), indicating that these cells escaped Cre-mediated removal of the β-catenin gene. The absence of β-catenin-negative DT cells in the mutant kidney suggests that β-catenin is required for the formation of DT. Our result is consistent with a previous report that the distal segment of the SSB is exposed to a higher level of Wnt/β-catenin signaling than the rest of the SSB^23^. This is also supported by the fact that the β-catenin-bound enhancers identified in MNPs are active in the distal segment of the SSB^19^. Although the distal segments of the developing nephron may experience a higher level of β-catenin signaling, we found that constitutive activation of β-catenin signaling did not promote the formation of the distal segments. Instead, it blocked the proper patterning of all nephron segments (Fig. 6 and Figure S6). Taking into account that the β-catenin GOF mutant cells show persistent expression of *Pax2* and that they fail to activate *Hnf4a* expression (Fig. 7 and Figure S7), it appears that constitutive activation of β-catenin signaling in epithelialized nephron progenitors results in developmental arrest. Although both *Wnt4GFPcre* and *Osr2Cre* caused developmental arrest in the β-catenin GOF mutant kidneys, these two mutants showed some slight differences. While no Slc12a1+ LOH or Slc12a3+ DT cells were formed with *Wnt4GFPcre* (Fig. 6), a very few Slc12a1+ or Slc12a3+ cells were found with *Osr2Cre* (Figure S6). This may be due to the fact that the distal segment of the SSB can be targeted by *Wnt4GFPcre*, but not by *Osr2Cre*. It is likely that *Wnt4GFPcre*-mediated activation of a stable form of β-catenin in the distal segment of the SSB blocked the initial specification of DT cells. However, it is unclear why the specification of LOH cells was affected by *Wnt4GFPcre*, but not by *Osr2Cre*, despite the fact that LOH cells are derived from the medial segment which is targeted by both Cre lines. It is possible that the development of one nephron segment (e.g. LOH) partially depends on another nephron segment (e.g. DT).

Constitutive activation of Wnt/β-catenin signaling is not compatible with proper nephrogenesis. Expression of a stable form of β-catenin by *Six2Cre*^17^, *Wnt4Cre*, or *Osr2Cre* blocks nephrogenesis. Although apparent phenotypes may look similar in these β-catenin GOF mutant kidneys, they show different points of developmental arrest in nephrogenesis. We have previously shown that, when *Six2Cre* is used, the arrest occurs at the pretubular aggregate stage^17^. In contrast, when *Wnt4Cre* or *Osr2Cre* is used, the developmental arrest occurs at the epithelial nephron progenitors (Fig. 7 and Figure S7). The fact that constitutive activation of β-catenin signaling causes arrest in nephrogenesis suggests that turning off the Wnt/β-catenin signaling is also critical for nephron formation. Taken together, it appears likely that transient activation of Wnt/β-catenin signaling is required at multiple steps of nephrogenesis.

Since β-catenin participates in not only canonical Wnt signaling but also cell adhesion, we cannot rule out the possibility that the phenotypes in β-catenin LOF and GOF mutant kidneys presented here are partly due to defective cell adhesion. Further investigation will be required to address this issue properly. One way is to test if deletion of genes encoding other components of the canonical Wnt pathway (such as Lrp5/Lrp6 or Tcf genes) recapitulates the β-catenin LOF mutant phenotypes. Alternatively, manipulation of genes encoding other adhesion factors would help resolve this issue. Nonetheless, considering the critical roles of Wnt/β-catenin signaling on cell fate determination in various biological contexts^14,15,50^, our genetic analyses of β-catenin is likely to represent the role of Wnt/β-catenin signaling in nephron segmentation. Overall, our data presented here suggest that β-catenin regulates the development of multiple nephron segments along the proximo-distal axis. It is likely that precise spatiotemporal activation and inactivation of β-catenin signaling is required for the proper formation of the mammalian nephron.

## Methods

### Mouse strains

All animal procedures were approved by the Institutional Animal Care and Use Committee at Cincinnati Children’s Hospital Medical Center in accordance to their guidelines for the care and use of laboratory animals (IACUC2017–0037). *Gt(ROSA)26Sor*^*tm3(CAG-EYFP)Hze*^ (*Rosa26*^*Ai3*^)^32^, *Wnt4*^*tm3(EGFP/cre)Amc*^ (*Wnt4GFPcre*)^24^, *Osr2*^*tm2(cre)Jian*^ (*Osr2Cre or Osr2*^*IresCre*^)^31^, *Ctnnb1*^*tm2Kem*^ (*Ctnnb1*^*c*^)^51^, and *Ctnnb1*^*tm1Mmt*^ (*Ctnnb1*^*ex3*^)^40^ mice were described previously.

### Immunofluorescence

Embryonic kidneys at E18.5 were fixed with 4% paraformaldehyde in PBS at room temperature for 10 min, incubated in 10% sucrose in PBS at 4 °C overnight, and imbedded in OCT. Cryosections of 10 or 12 μm were incubated in PBS containing 0.1% Triton x-100, 5% heat-inactivated sheep serum, and the following antibodies: Jag1 (TS1.15 H, rat, 1:20, Developmental Studies Hybridoma Bank), Wt1 (sc-7385, mouse IgG1, 1:100, Santa Cruz), GFP (GFP-1020, chick IgY, 1:500, Aves Labs), Slc12a3 (HPA028748, rabbit, 1:300, Sigma), LTL (FL-1321, FITC, 1:200, Vector Laboratories), Slc12a1 (18970–1-AP, rabbit, 1:200, Proteintech), β-catenin (71–2700, rabbit, 1:500, Invitrogen), β-catenin (sc-7963, mouse IgG1, 1:50, Santa Cruz), Pecam1 (sc-18916, rat, 1:300, Santa Cruz), Mafb (HPA005653, rabbit, 1:300, Sigma), Akap12 (25199–1-AP, rabbit, 1:300, Proteintech), Cdh6 (HPA007047, rabbit, 1:300, Sigma), Hnf4a (ab41898, mouse IgG2a, 1:500, Abcam), Pax2 (21385–1-AP, rabbit, 1:200, Proteintech), Slc5a2 (HPA041603, rabbit, 1:100, Sigma) Fluorophore-conjugated secondary antibodies (Thermo Fisher Scientific or Jackson ImmunoResearch Laboratories) were used at a 1:500 dilution. The Jag1 antibody developed by Spyros Artavanis-Tsakonas was obtained from the Developmental Studies Hybridoma Bank, created by the NICHD of the NIH and maintained at The University of Iowa, Department of Biology, Iowa City, IA 52242. Nuclei were stained with Hoechst 33342 before mounting. Images were acquired on a Nikon TiE microscope with Andor Zyla 4.2 camera and Lumencor Spectra X light source housed at the Confocal Imaging Core at CCHMC.

## Supplementary information

Supplementary figures and legends

## Data Availability

Data will be made available upon request.
