# Supplementary material for: β-catenin regulates the formation of multiple nephron segments in the mouse kidney
Source: Sci Rep. 2019 Nov 4;9:15915. doi: 10.1038/s41598-019-52255-w (PMC6828815; doi:10.1038/s41598-019-52255-w)
Supplement: Supplementary file 1 — Supplementary figures and legends [file 41598_2019_52255_MOESM1_ESM.pdf]

Title Page

Title: β-catenin regulates the formation of multiple nephron segments in the mouse kidney

Patrick Deacon,[1] Charles W. Concodora,[1,2] Eunah Chung, and Joo-Seop Park[*]

Division of Pediatric Urology and Division of Developmental Biology, Cincinnati Children's Hospital Medical Center, Cincinnati, OH 45229, USA

University of Cincinnati College of Medicine, Cincinnati, OH 45267, USA

[1]These authors contributed equally to this work.

[2]Current address: Urology for Children, 200 Bowman Drive, Voorhees, NJ 08043, USA

[*]Corresponding author:

Joo-Seop Park
Cincinnati Children's Hospital Medical Center
Location R1566, ML7007
3333 Burnet Avenue
Cincinnati, OH 45229
TEL: 513-803-7871
FAX: 513-636-4317
Email: joo-seop.park@cchmc.org

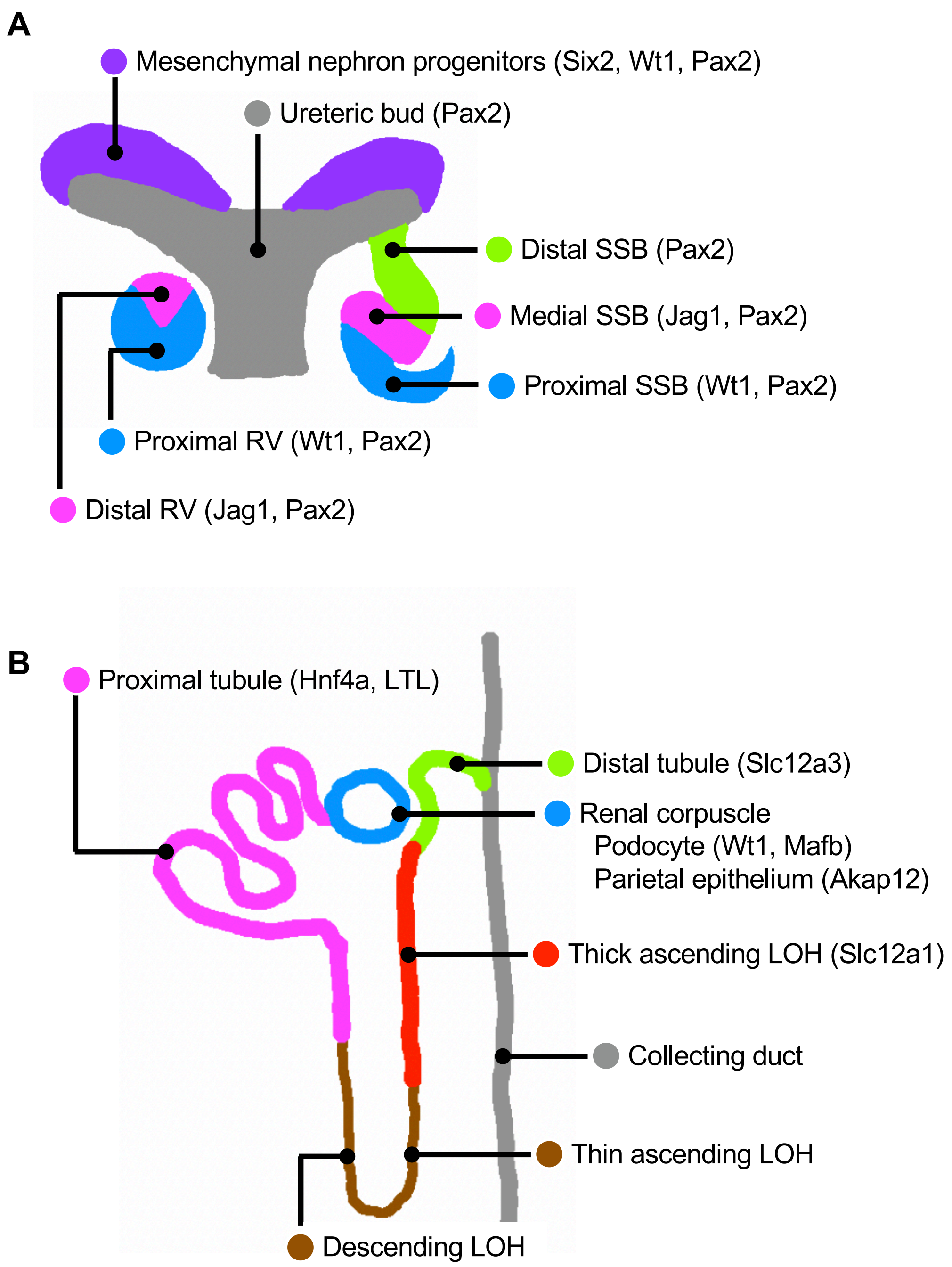

Figure S1. Schematic illustrations of distinct segments and their markers in parentheses during nephron development. (A) Mesenchymal nephron progenitors become epithelialized to form the renal vesicle (RV), which further develops into the S-shaped body (SSB). (B) Segments of the fully formed nephron.

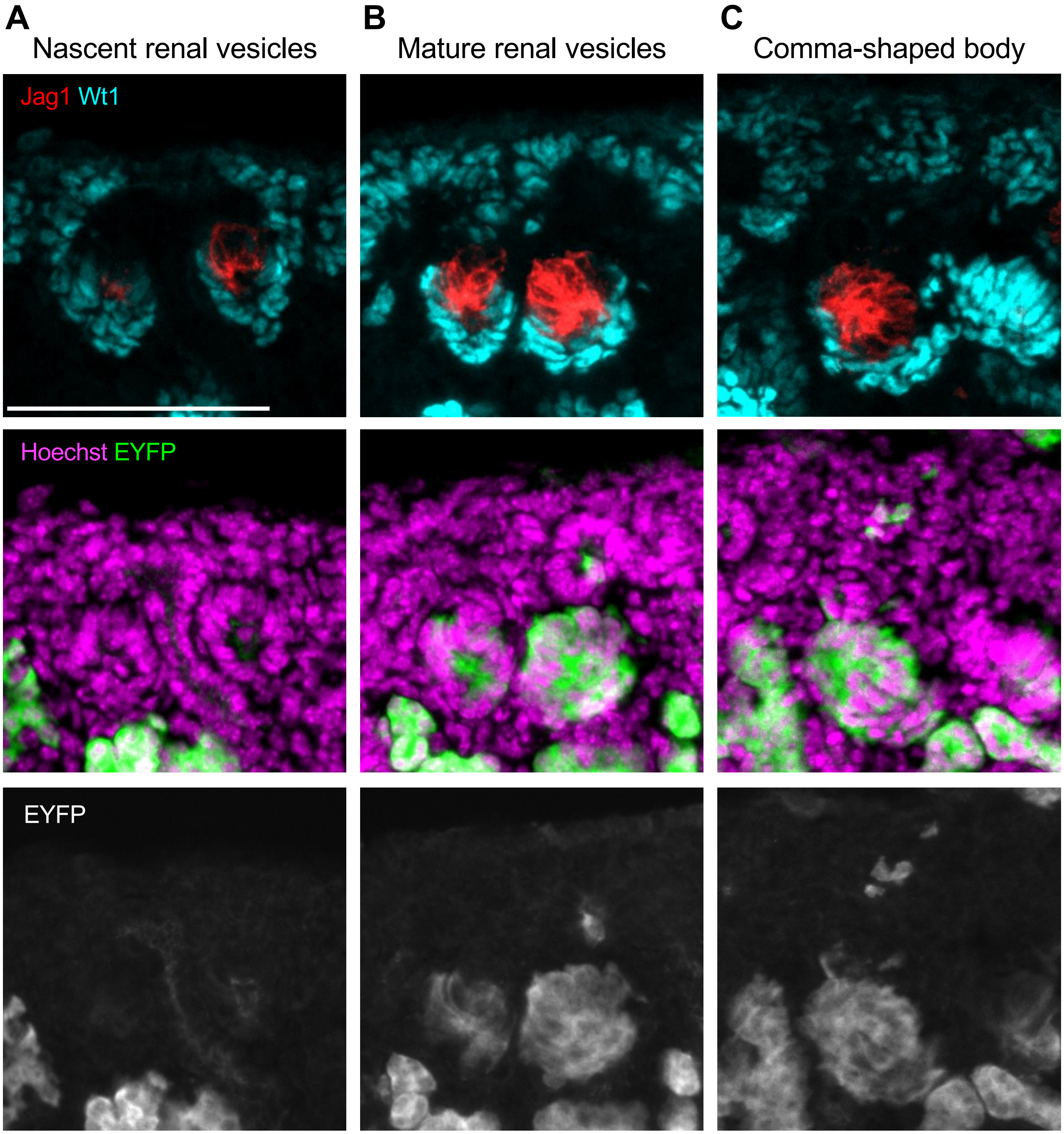

Figure S2. *Osr2Cre* activity in the renal vesicle and its derivatives. In the mouse embryonic kidney (*Rosa26*$^{Ai3/+}$;*Osr2*$^{IresCre/+}$), *Osr2Cre* activity was monitored by the activation of the Rosa reporter (EYFP). The Rosa reporter was inactive in nascent renal vesicles (A) but active in mature renal vesicles (B) and the comma-shaped body (C). Mature renal vesicles express Jag1 and Wt1 at higher levels than nascent renal vesicles. Stage E18.5. Scale bar: 100μm.

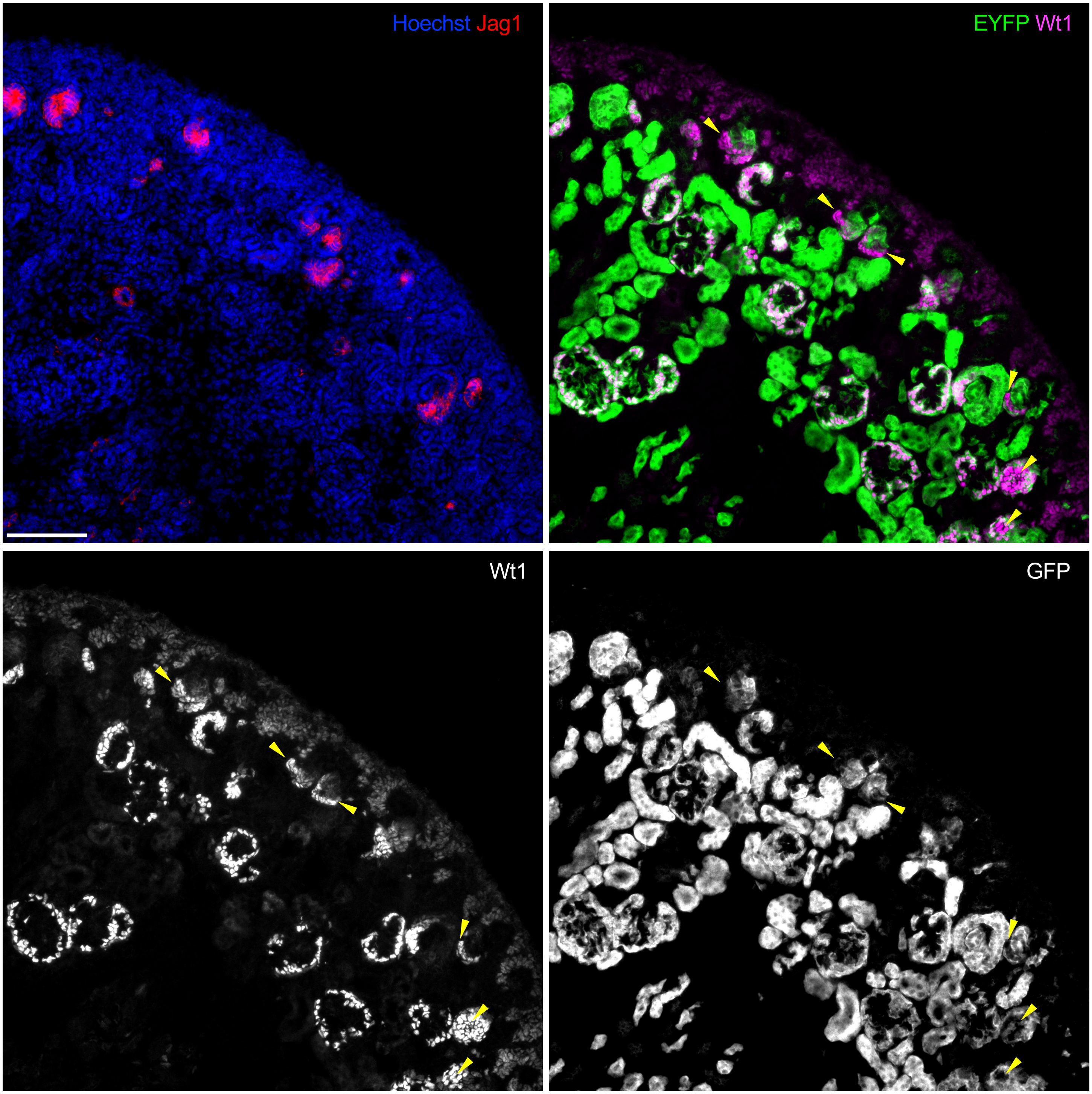

Figure S3. *Osr2Cre* mosaically targets Wt1+ cells in the nascent nephrons. In the nephrogenic zone of the mouse embryonic kidney (*Rosa26*$^{Ai3/+}$*;Osr2*$^{IresCre/+}$), a subset of Wt1+ cells were not labeled with the Rosa reporter (yellow arrowheads). However, outside of the nephrogenic zone, most of the Wt1+ cells in glomeruli were labeled with the Rosa reporter. Stage E18.5. Scale bar: 100μm.

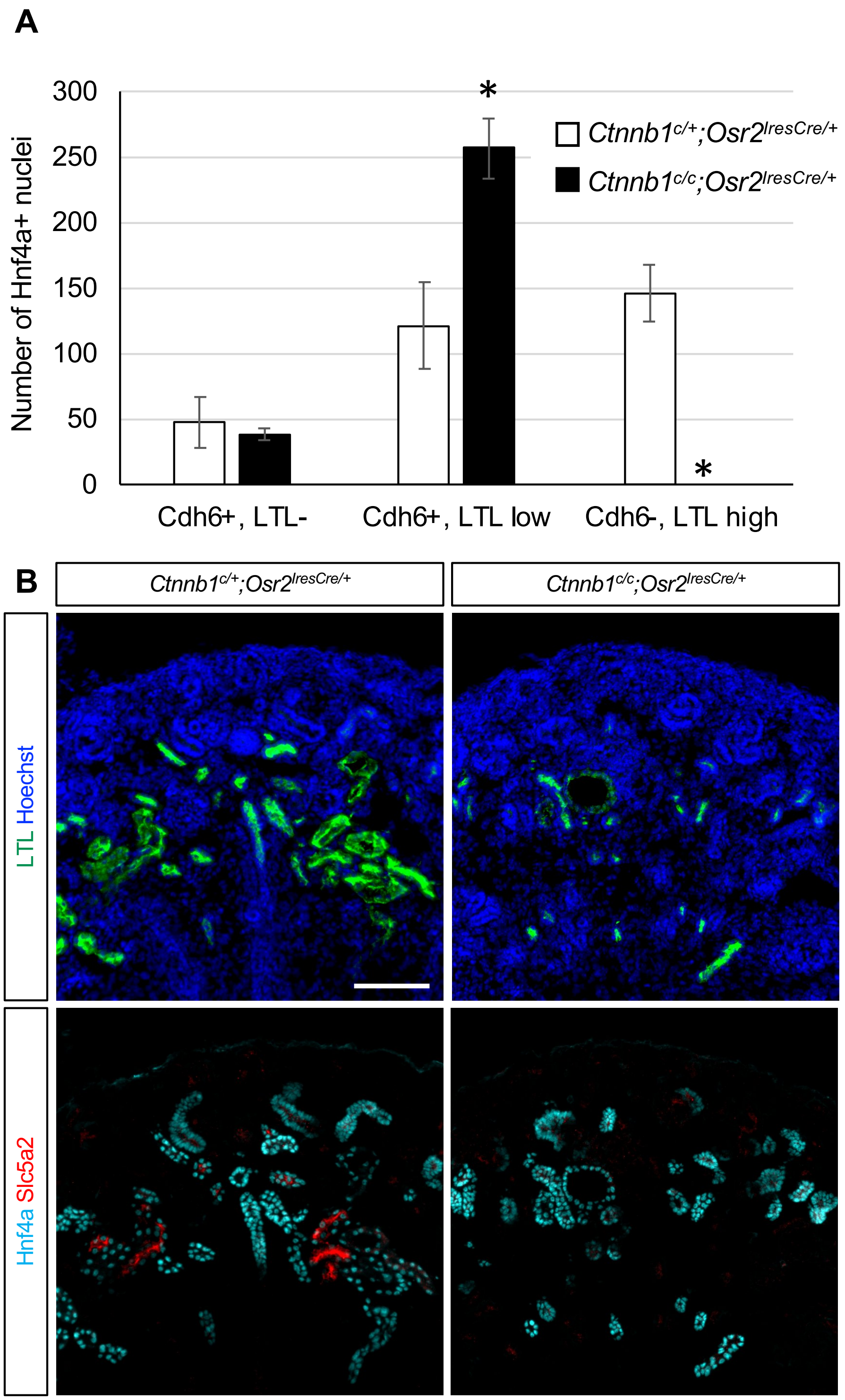

Figure S4. Proximal tubule development is defective in the β-catenin loss-of-function mutant kidney. (A) Quantification of proximal tubule development in the β-catenin loss-of-function mutant kidney by *Osr2Cre.* Based on Cdh6 and LTL staining as shown in Figure 4, Hnf4a+ nuclei were placed into three groups. The mutant kidneys have more Cdh6+ LTL-low cells than the control kidney. Cdh6-negative LTL-high cells are absent in the mutant kidney. **p* < 0.01 as determined by two-tailed Student's *t* test. Error bars represent standard deviation. (B) The sodium-glucose cotransporter Slc5a2 is downregulated in the β-catenin loss-of-function mutant kidney by *Osr2Cre*. In the control kidney, Slc5a2 is detected in a subset of mature proximal tubule cells. In contrast, Slc5a2 is absent in the mutant kidney. Scale bar: 100μm.

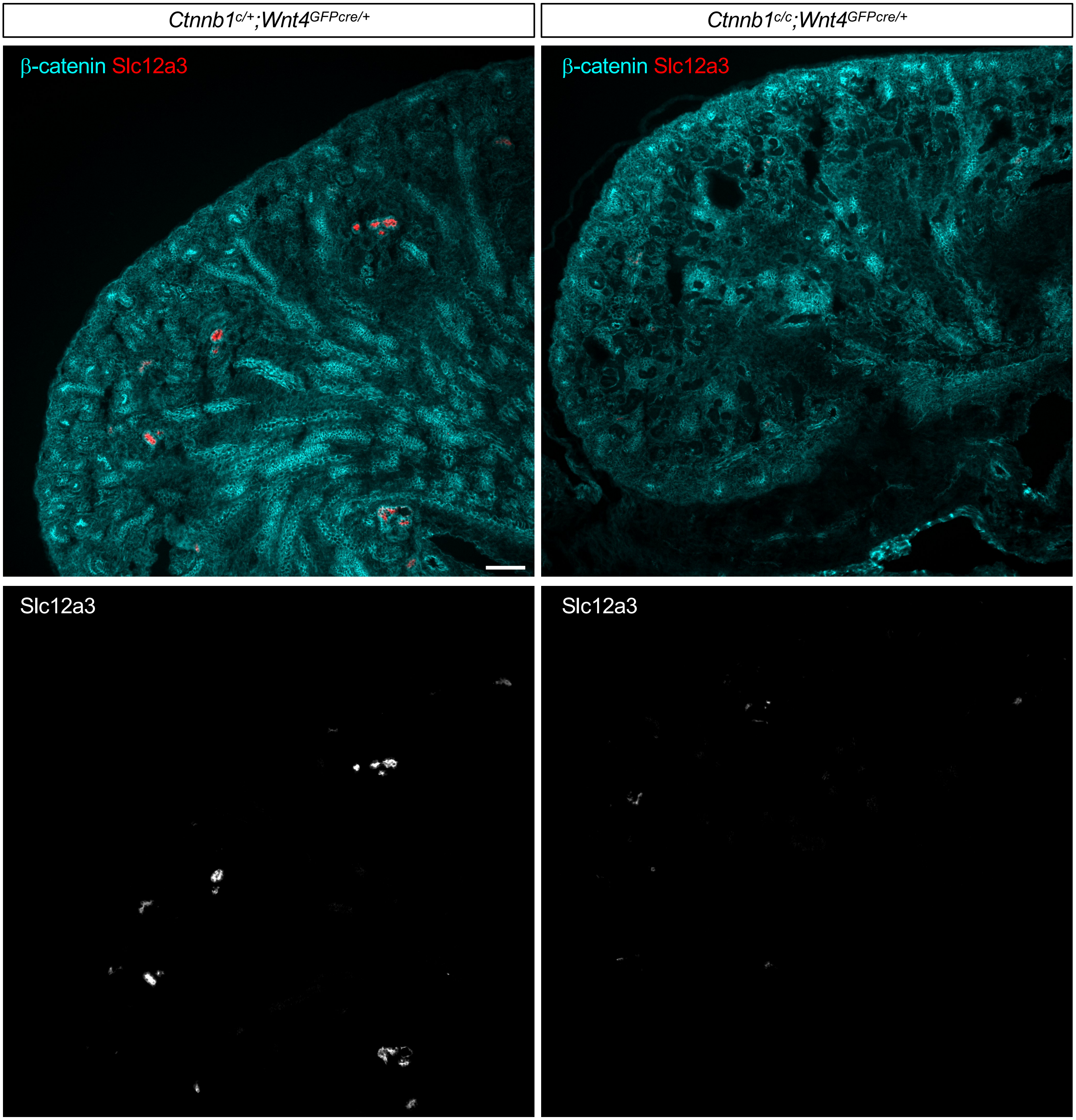

Figure S5. Expression of Slc12a3, a distal tubule marker, is considerably reduced in the β-catenin loss-of-function mutant kidney by *Wnt4GFPcre*. In the mutant kidney, the few detectable Slc12a3+ distal tubules are positive for β-catenin, suggesting that these cells have escaped *Wnt4GFPcre*-mediated removal of β-catenin. Consistent with this, our RT-qPCR analysis showed that the amount of Slc12a3 transcripts in the mutant kidney is 32% that of the control kidney (average fold expression=0.3249, stdev=0.04415, n=4). Stage E18.5. Scale bar: 100μm.

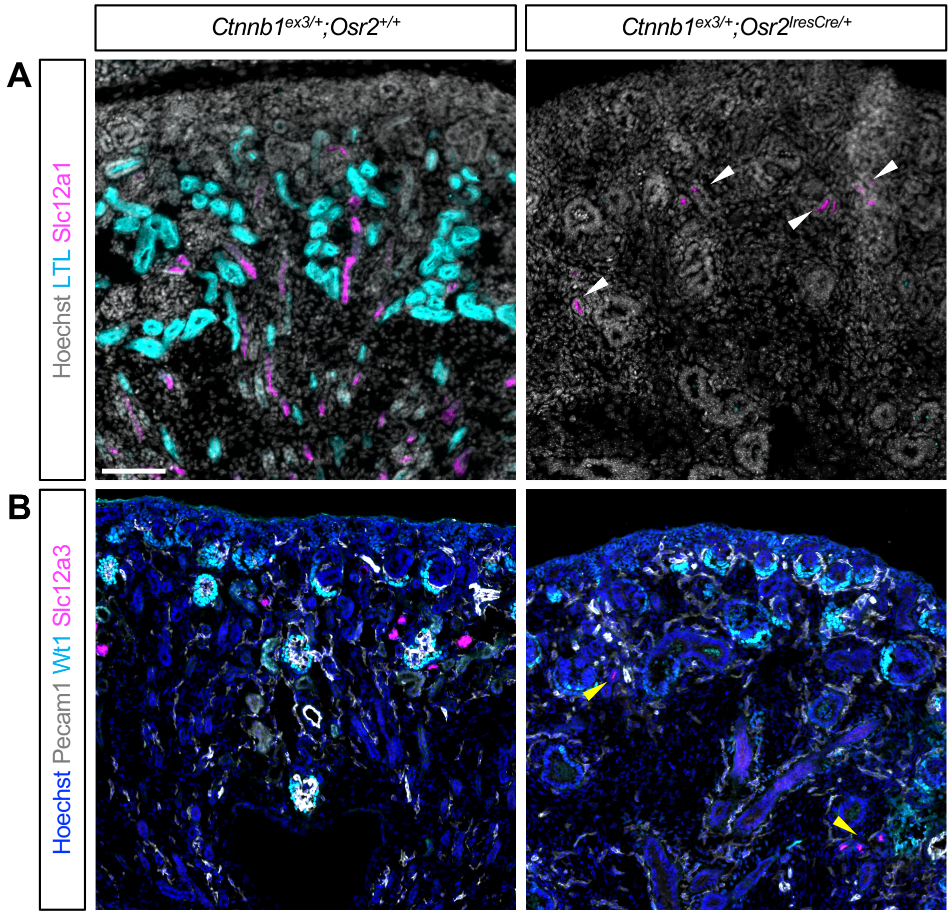

Figure S6. Expression of a stable form of β-catenin inhibits proper nephron patterning. The β-catenin gain-of-function mutant kidney by *Osr2Cre* fails to form properly patterned nephrons. No proximal tubules (A) or glomeruli (B) are formed in the mutant. Slc12a1+ loop of Henle (marked by white arrowheads, A) or Slc12a3+ distal tubule (marked by yellow arrowhead, B) cells are present but they fail to elongate in the mutant. Stage E18.5. Scale bar: 100μm.

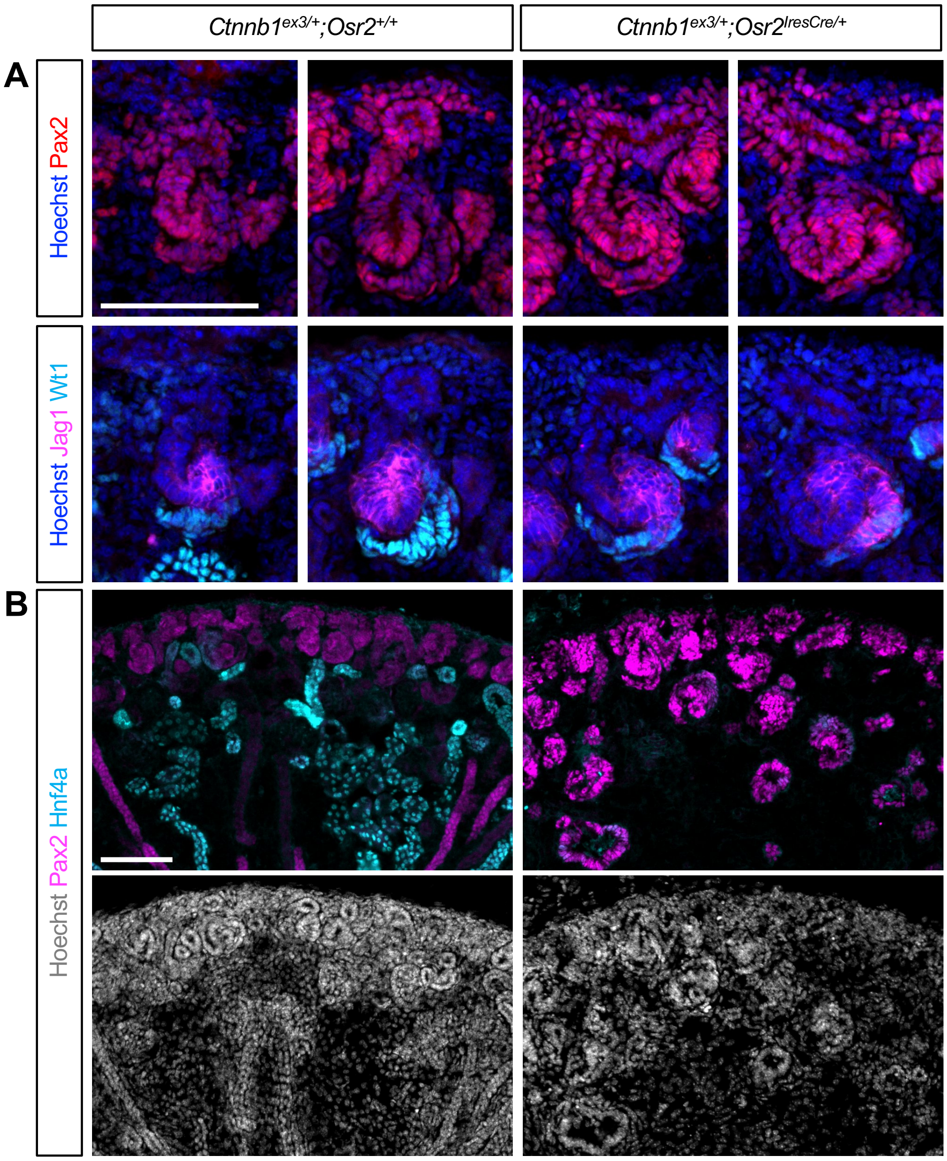

Figure S7. Expression of a stable form of β-catenin prevents epithelial nephron progenitors from further differentiating. (A) Nascent (left) and mature (right) S-shaped bodies are shown for each genotype. In the control kidney, Jag1 and Wt1 mark the medial and proximal segments of the S-shaped body, respectively. In the β-catenin gain-of-function mutant kidney by *Osr2Cre*, the Jag1 expression domain expands into the proximal segment of the S-shaped body. Pax2 marks the collecting duct, the cap mesenchyme, and nascent developing nephrons. (B) In the control kidney, Pax2 expression in epithelial nephron progenitors is downregulated after S-shaped body stage. As a result, most of the Hnf4a+ cells are negative for Pax2. In the β-catenin gain-of-function mutant kidney by *Osr2Cre,* Pax2 is persistently expressed in the nephron lineage and little Hnf4a expression is detected, suggesting that β-catenin gain-of-function mutant cells fail to exit from their progenitor status. Stage E18.5. Scale bar: 100μm.
